# Antimicrobial activity of photosensitizers: arrangement in bacterial membrane matters

**DOI:** 10.3389/fmolb.2023.1192794

**Published:** 2023-05-15

**Authors:** Oleg V. Batishchev, Maksim A. Kalutskii, Ekaterina A. Varlamova, Anna N. Konstantinova, Kirill I. Makrinsky, Yury A. Ermakov, Ivan N. Meshkov, Valerij S. Sokolov, Yulia G. Gorbunova

**Affiliations:** ^1^ Frumkin Institute of Physical Chemistry and Electrochemistry, Russian Academy of Sciences, Moscow, Russia; ^2^ Kurnakov Institute of General and Inorganic Chemistry, Russian Academy of Sciences, Moscow, Russia

**Keywords:** photosensitizer, antimicrobial photodynamic therapy, lipid membrane, antibiotic, molecular dynamics simulation, *Echerichia coil*, *Acinetobacter baumanii*, membrane potentials

## Abstract

Porphyrins are well-known photosensitizers (PSs) for antibacterial photodynamic therapy (aPDT), which is still an underestimated antibiotic-free method to kill bacteria, viruses, and fungi. In the present work, we developed a comprehensive tool for predicting the structure and assessment of the photodynamic efficacy of PS molecules for their application in aPDT. We checked it on a series of water-soluble phosphorus(V) porphyrin molecules with OH or ethoxy axial ligands and phenyl/pyridyl peripheral substituents. First, we used biophysical approaches to show the effect of PSs on membrane structure and their photodynamic activity in the lipid environment. Second, we developed a force field for studying phosphorus(V) porphyrins and performed all-atom molecular dynamics simulations of their interactions with bacterial lipid membranes. Finally, we obtained the structure-activity relationship for the antimicrobial activity of PSs and tested our predictions on two models of Gram-negative bacteria, *Escherichia coli* and *Acinetobacter baumannii*. Our approach allowed us to propose a new PS molecule, whose MIC_50_ values after an extremely low light dose of 5 J/cm^2^ (5.0 ± 0.4 μg/mL for *E. coli* and 4.9 ± 0.8 μg/mL for *A. baumannii*) exceeded those for common antibiotics, making it a prospective antimicrobial agent.

## Introduction

The World Health Organization (WHO) stated several years ago that we have come to the post-antibiotic era because of the growing number of bacteria that are resistant to all known antibiotics (World Health Organization, 2014). Drug-resistant bacteria are currently responsible for over 700,000 global deaths annually, and their mortality is predicted to exponentially rise to above 10 million deaths per year by 2050 (Renwick et al., 2016). The rapid increase in the number of multidrug-resistant bacteria, especially during the COVID-19 pandemic and the significant unmanaged use of antibacterial substances, urgently requires new weapons to be used in the fight against infections (Lai et al., 2021). At the same time, the rate of introduction of new antibiotics has diminished in the last decade. The benefits of novel antibiotics are limited by the appearance of new resistive strains during the first year of clinically approved treatment (Spellberg et al., 2008; Tängdén et al., 2018).

Antibacterial photodynamic therapy (aPDT) is an antibiotic-free method of bacterial inactivation (Jori and Brown, 2004; Jori et al., 2006; Caminos et al., 2008; Liu et al., 2015; da Fonseca et al., 2021), which can be also applied to suppress the activity of viruses, fungus, and protozoa (Aroso et al., 2021; Zhu et al., 2021; Ziental et al., 2021; Duguay et al., 2022). The method implies the application of photosensitizers (PSs), which provide photoinduced generation of reactive oxygen species (ROS) including singlet oxygen. They irreversibly damage the structural elements of bacterial cells and induce their death (Merchat et al., 1996; Minnock et al., 1996). In early studies, these PSs were phenothiazine derivatives, methylene blue, and toluidine blue, known from the 19th century. These are the few dyes currently approved for clinical practice (Cieplik et al., 2017; Wainwright et al., 2017). The main mechanism of their photodynamic activity is a generation of ROS, which cause cellular death because of oxidative stress. However, bacteria have enzymatic systems that can neutralize ROS to some extent, decreasing the efficacy of aPDT treatment (Kim et al., 2001). Several studies based on the luminescence of ROS in cells demonstrate the major role of singlet oxygen in photodynamic damage of the cell (Jones and Grossweiner, 1994; Kilger et al., 2001). Measurements of time-decay luminescence reveal that the photodynamic action of PS molecules significantly depends on oxygen supply, which is in the case of bacteria accumulated predominantly in the outer cell wall areas or in adjacent cytoplasmic membranes (Maisch et al., 2007). Therefore, PSs located in the membrane and producing singlet oxygen should have maximal antimicrobial activity.

For the moment, application of aPDT is limited mainly by dentistry and skin infections, because it requires delivery of light to the exact part of the human body, which is not possible for every wavelength. Despite the development of fiber optics, allowing light delivery to almost every part of the human body, wide usage of aPDT is still hampered by their poor water solubility, photostability, and, more importantly, lack of selectivity towards target organisms. All of these disadvantages demand a better understanding of the molecular mechanisms of PS activity in bacteria. Investigations of aPDT mechanisms evidence that the targets of oxidative stress can be different. The damage can occur at the surface of the cell or in its inner structures (cytoplasmic proteins, DNA, etc.) (Alves et al., 2014). Recent studies suggest that the accumulation of PS in a lipid membrane should prevail in their ability to kill bacteria (Almeida et al., 2015). Localization of PS molecules in bacteria depends on their chemical structure (molecular mass, charge, lipophilic/hydrophilic balance, etc.), thus, establishing the correlation between the structure of the molecule and its aPDT activity is an important task. It requires understanding the activity of photosensitizers in the membrane environment rather than in a bulk organic phase.

Among photosensitizers generating singlet oxygen, special attention is paid to tetrapyrrolic macrocycles of both natural and synthetic origin (Nyokong and Ahsen, 2012; Ghazal et al., 2017; Mesquita et al., 2018; Nyamu et al., 2018; Otvagin et al., 2019). Unsubstituted porphyrins and phthalocyanines are hydrophobic, therefore, much effort has been made to designing water-soluble compounds, achieved either by the introduction of neutral hydrophilic, or ionogenic groups (Singh et al., 2015; Kollar et al., 2020). Thus, the introduction of quaternary ammonium or pyridinium groups afforded cationic PS, while the introduction of carboxylates, phosphonates, or sulfo-groups resulted in the formation of anionic PS (Dumoulin et al., 2010). In general, cationic PS are more efficient agents for aPDT in comparison with neutral and anionic molecules, because cations are able to bind to negatively charged lipopolysaccharide groups of Gram-negative bacteria and subsequently penetrate into the lipid matrix of their membranes (Ball et al., 1999; Tim, 2015; Ghazal et al., 2017; Miretti et al., 2017). This makes cationic PS more versatile agents for aPDT, in contrast to anionic PSs, which are less active against Gram-positive bacteria (Ghorbani et al., 2018), but increases their dark toxicity and production costs (Liu et al., 2015).

The WHO suggested paying special attention to multidrug-resistant tuberculosis and Gram-negative bacteria because of their substantial morbidity and mortality (Tacconelli et al., 2018). Among Gram-negative bacteria, *Escherichia coli* appeared to be one of the most common causes of antibiotic-resistant infections (Jones, 2001). Another dangerous threat is *Acinetobacter baumannii*, which demonstrates a perfect ability to form biofilms and thus enhance its multidrug resistance (Dijkshoorn et al., 2007; Ibrahim et al., 2021).

We have shown recently that the photodynamic efficacy of both substituted porphyrins and phthalocyanines depends on their ability to bind to lipid membranes (Sokolov et al., 2018; Jiménez-Munguía et al., 2019). We have suggested a model describing the effect of PS location in the membrane on its ability to generate singlet oxygen. The approach we have utilized to study the binding of PS to biological membranes is *in vitro* modeling of photodynamic processes using model bilayer lipid membranes (BLM) (Konstantinova et al., 2018; Sokolov et al., 2018; Jiménez-Munguía et al., 2019; Polivanovskaia et al., 2022). Simultaneously, we have demonstrated that cationic phosphorus(V) porphyrins possess remarkable photophysical properties including efficient generation of singlet oxygen in water that can be tuned by the varying nature of axial ligands (Meshkov et al., 2017).

**CHART 1:**
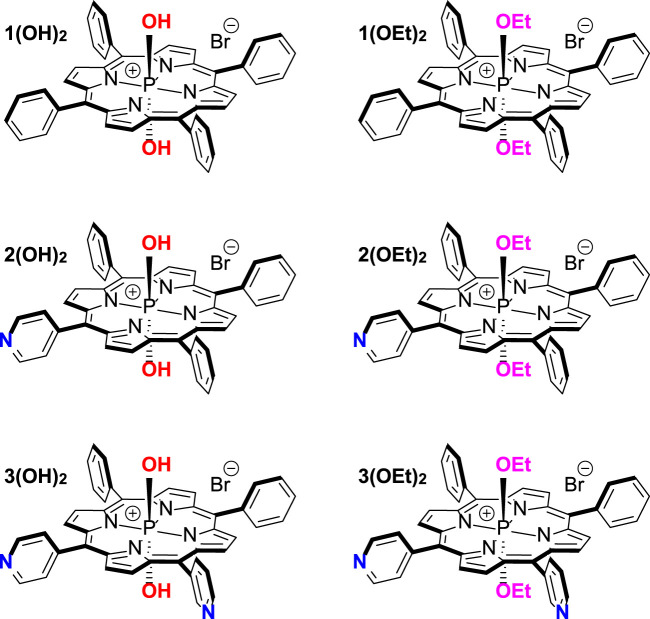
Structures of investigated phosphorus(V) porphyrins.

In the present work, we aimed to clarify the main molecular features determining PS efficacy as an aPDT agent. We performed biophysical investigations of adsorption, penetration, and photodynamic efficiency at model lipid membranes for three series of phosphorus(V) porphyrins bearing two axial ligands. These compounds are: dihydroxido (5,10,15,20-tetraphenylporphyrinato)phosphorus(V) bromide **1(OH)**_**2**_, diethoxido (5,10,15,20-tetraphenylporphyrinato)phoshоrus(V) bromide **1(OEt)**_**2**_, dihydroxido (5-pyridyl-(10,15,20-triphenyl)porphyrinato)phosphorus(V) bromide **2(OH)**_**2**_, diethoxido (5-pyridyl-(10,15,20-triphenyl)porphyrinato)phosphorus(V) bromide **2(OEt)**_**2**_, dihydroxido (5,10-dipyridyl-(15,20-diphenyl)porphyrinato)phosphorus(V) bromide **3(OH)**_**2**_, and diethoxido (5,10-pyridyl (15,20-diphenyl)porphyrinato)phosphorus(V) bromide **3(OEt)**_**2**_ (Chart 1). We developed a force field for all of these PS molecules and performed all-atom molecular dynamics simulations of their membrane binding and arrangement in the lipid bilayer. Based on obtained results we suggested both a new perspective molecule for aPDT as well as a relation between the structure and bactericidal activity of phosphorus(V) porphyrins. These predictions were tested on antibacterial activity on two models of Gram-negative bacteria, *E. coli* and *A. baumannii*, and demonstrated a good relationship between the membrane arrangement of the PS molecule and the best minimal inhibitory concentration, MIC_50_. Our approach manifested a general interconnection between PS position and orientation in the membrane and its antimicrobial activity, thus providing a new way for both designing and testing PS molecules for aPDT.

## Materials and methods

### Synthesis and characterization of porphyrins

Solvents were dried using standard techniques: CH_2_Cl_2_, CHCl_3,_ and pyridine were distilled over CaH_2_. All air and water-sensitive experiments were carried out under an argon atmosphere using standard vacuum line techniques. All chemicals were obtained commercially and used without further purification. ^1^H, ^13^C, and ^31^P NMR spectra were acquired on a Bruker AV 600 (600 MHz) spectrometer, with a deuterated solvent as the lock and residual solvent as the internal reference. Absorption spectra were recorded using an Evolution 210 spectrophotometer (Thermo Scientific). MicroTOF-Q LC (Bruker Daltonics, Bremen) spectrometer equipped with an electrospray source was used for the high-resolution electrospray mass spectrometry measurements (HR ESI-MS). BioRad Bio Beads S-X1 and S-X3 gels were used for the gel-permeation chromatography (GPC).

Free-base porphyrins **1-3** were prepared following the standard procedures (Adler et al., 1967; Barton et al., 2000). Phosphorus (V) complexes with porphyrins **1** and **2** were synthesized using previously reported methods (Meshkov et al., 2016; Meshkov et al., 2017).

**Compound 3(OH)**_**2**_. The 5,10-dipyridyl (15,20-diphenyl)porphyrin (56 mg, 9.08 × 10^−5^ mol, 1 equiv) was dissolved in pyridine (40 mL) under argon and then a solution of POBr_3_ (1.38 g, 4.81 mmol, 53 equiv) in pyridine (10 mL) was added dropwise under stirring. The reaction mass was refluxed for 2 h under argon, cooled to room temperature, and poured into the mixture of water (1 L) and DCM (200 mL). The reaction mass was stirred for 48 h at room temperature, then the organic layer was isolated and washed with distilled water (5 × 500 mL). Next, the purple organic layer was diluted with 100 mL of *n*-hexane and poured on a silica gel chromatography column without evaporation of solvents. Increasing the polarity of the eluent (DCM-methanol) up to 15% of MeOH gave a crude product that was subsequently purified on Bio-Beads S-X3 (eluent - chloroform-methanol 95:5) and Bio-Beads S-X1 (eluent - chloroform-methanol 97.5:2.5) columns affording 40 mg of the pure compound **3(OH)**_**2**_ as a purple solid in 53% yield. ^1^H NMR (CDCl_3_+CD_3_OD, 600 MHz): δ 7.67 (m, 6H CH_Ph meta/para_), 7.91 (d, ^3^ *J* = 7.2 Hz, 4H, CH_Ph ortho_), 7.91 (d, ^3^ *J* = 7.2 Hz, 4H, CH_Ph ortho_), 7.96 (d, ^3^ *J* = 5.0 Hz, 4H, CH_Py ortho_), 8.82 (m, 6H, CH_Py ortho/β-pyrrolic_), 8.85 (d, ^4^ *J*_*P-H*_ = 2.4 Hz, 2H, CH _β-pyrrolic_), 8.90 (d, ^4^ *J*_*P-H*_ = 2.4 Hz, 2H, CH _β-pyrrolic_), 8.93 (dd, ^3^ *J* = 5.3 Hz, ^4^ *J*_*P-H*_ = 2.4 Hz, 2H, CH _β-pyrrolic_). ^31^P NMR (CDCl_3_+CD_3_OD, 243 MHz): δ −191. ^13^C NMR (CD_3_OD, 151 MHz): δ 113.2 (C), 118.0 (C), 129.2 (CH), 129.8 (CH), 130.7 (CH), 133.5 (CH, ^3^ *J*_*P-C*_ = 4.7 Hz), 133.9 (CH, ^3^ *J*_*P-C*_ = 4.7 Hz), 134.5 (CH, ^3^ *J*_*P-C*_ = 4.7 Hz), 134.6 (CH), 134.9 (CH, ^3^ *J*_*P-C*_ = 4.7 Hz), 137.4 (C), 139.4 (C), 139.5 (C), 140.5 (C), 140.6 (C), 146.6 (C), 150.0 (CH). λ max (nm) (logε, mol^−1^ L cm^−1^) 427 (5.24), 553 (4.05), 589 (3.35). HR-ESI MS: *m/z* obsd 340.1039, calcd 340.1039 [(M + H-Br)^2+^]; obsd 679.2006, calcd 679.2006 [(M-Br)^+^]; M = C_42_H_28_BrN_6_O_2_P.

**Compound 3(OEt)**_**2**_. The 5,10-dipyridyl (15,20-diphenyl)porphyrin (60 mg, 9.73 × 10^−5^ mol, 1 equiv) was dissolved in pyridine (40 mL) under argon and then a solution of POBr_3_ (1.48 g, 5.15 mmol, 53 equiv) in pyridine (10 mL) was added dropwise under stirring. The reaction mass was refluxed for 2 h under argon, cooled to room temperature, and diluted with 300 mL of ethanol. The reaction mass was stirred for 48 h at room temperature, then the organic layer was diluted with 300 mL of DCM, isolated, and washed with distilled water (5 × 500 mL). Next, the violet organic layer was diluted with 200 mL of *n*-hexane and poured on a silica gel chromatography column without evaporation of solvents. Increasing the polarity of the eluent (DCM-methanol) up to 12% of MeOH gave a crude product that was subsequently purified on Bio-Beads S-X3 (eluent—chloroform-methanol 95:5) and Bio-Beads S-X1 (eluent–chloroform-methanol 98:2) columns affording 12 mg of the pure compound **3(OEt)**_**2**_ as a purple solid in 15% yield. ^1^H NMR (CD_3_OD, 600 MHz): δ −2.26 (dq, ^3^ *J*_*P-H*_ = 13.6 Hz, ^3^ *J* = 6.8 Hz, 4H, CH_2_), −1.76 (td, ^3^ *J* = 7.2 Hz, ^4^ *J*_*P-H*_ = 1.9 Hz, 6H, CH_3_), 7.84 (m, 6H, CH_Ph meta/para_), 8.05 (d, ^3^ *J* = 6.9 Hz, 4H, CH_Ph ortho_), 8.15 (d, ^3^ *J* = 5.4 Hz, 4H, CH_Py ortho_), 9.02 (d, ^3^ *J* = 5.4 Hz 4H CH_Py meta_), 9.20 (m, 8H, CH_β-pyrrolic_). ^31^P NMR (CD_3_OD, 121 MHz): δ −179. ^13^C NMR (CD_3_OD, 151 MHz): δ 13.4 (CH_3_, ^3^ *J*_*P-C*_ = 17.1 Hz), 58.1 (CH_2_, ^2^ *J*_*P-C*_ = 15.2 Hz), 113.9 (C_meso(Py)_, ^3^ *J*_*P-C*_ = 1.8 Hz), 118.6 (C_meso(Ph)_, ^3^ *J*_*P-C*_ = 1.8 Hz), 129.5 (CH_Ph_), 129.8 (CH_Ph_), 131.0 (CH_Py ortho_), 134.0 (CH_β-pyrrolic_, ^3^ *J*_*P-C*_ = 5.2 Hz), 134.4 (CH_β-pyrrolic_, ^3^ *J*_*P-C*_ = 5.2 Hz), 134.7 (CH_Ph ortho_), 135.0 (CH_β-pyrrolic_, ^3^ *J*_*P-C*_ = 5.2 Hz), 135.3 (CH_β-pyrrolic_, ^3^ *J*_*P-C*_ = 5.2 Hz), 136.8 (C_Ph_, ^4^ *J*_*P-C*_ = 0.4 Hz), 139.8 (C_α-pyrrolic_, ^2^ *J*_*P-C*_ = 0.8 Hz), 139.9 (C_α-pyrrolic_, ^2^ *J*_*P-C*_ = 0.8 Hz), 140.9 (C_α-pyrrolic_, ^2^ *J*_*P-C*_ = 0.8 Hz), 141.0 (C_α-pyrrolic_, ^2^ *J*_*P-C*_ = 0.8 Hz), 146.9 (C_Py_, ^4^ *J*_*P-C*_ = 0.3 Hz), 150.3 (CH_Py meta_). UV-Vis (CHCl_3_) λ max (nm) (logε, mol^-1^ L cm^-1^) 430 (5.44), 559 (4.24), 595 (3.54). HR-ESI MS: *m/z* obsd 735.2622, calcd 735.2632 [(M-Br)^+^]; M = C_46_H_36_BrN_6_O_2_P.

The photophysical properties of **3(OH)**_**2**_ and **3(OEt)**_**2**_ were determined as described by Meshkov et al. (2017). In brief, fluorescence quantum yields (Φ_F_) were determined by a comparative method with 5,10,15,20-tetraphenylporphyrin in toluene (Φ_F_ = 0.11) (Dolphin, 1978) as a standard. For singlet oxygen quantum yield determination (Φ_Δ_), we utilized a previously developed experimental setup (Meshkov et al., 2017), and 5,10,15,20-tetraphenylporphyrin in chloroform (Φ_Δ_ = 0.50) (Schmidt et al., 1989) and 5,10,15,20-tetra(4-sulfonatophenyl)-porphyrin in water (Φ_Δ_ = 0.64) (Davila and Harriman, 1990) were used as a reference for organic solvent and water, respectively.

### Experiments on bilayer lipid membranes

The experimental setup was as described by us earlier (Sokolov and Pohl, 2009; Jiménez-Munguía et al., 2019). In brief, bilayer lipid membranes (BLMs) were formed by the Mueller-Rudin technique (Mueller et al., 1963) from a 15 mg/mL solution of 1,2-diphytanoyl-*sn*-glycero-3-phospholcholine, DPhPC (Avanti Polar Lipids, Alabaster, AL, United States), in *n*-decane (Sigma-Aldrich, Saint-Louis, MO, United States) at an aperture (diameter of 0.8 mm) in a septum dividing two compartments (2 mL each) of a Teflon cell. During the experiment, both compartments were continuously stirred by a magnetic stirrer. The cell was equipped with two windows: one for monitoring the formation of the BLM and the other for illumination of the membrane. Working buffer solutions were prepared with KCl (“Reachim,” Russia), and 4-(2-hydroxyethyl)-1-piperazineethanesulfonic acid, HEPES (Sigma-Aldrich, Saint-Louis, MO, United States), dissolved in double-distilled water. Porphyrins and the styryl dye 4-{2-[6-(Dibutylamino)-2-naphthalinyl]-ethenyl}-1-(3-sulfopropyl)-pyridiniumhydroxid, di-4-ANEPPS (Sigma-Aldrich, Saint-Louis, MO, United States) were added from stock solutions in ethanol. The total concentration of ethanol in water never exceeded 3%. In control experiments, we proved that such an amount of ethanol did not affect the stability of the BLM (data not shown). The stability of phosphorus(V) porphyrin complexes in the working buffer was evaluated by measuring the absorption spectra every 10 min for 1 h. Measurements were performed on Panorama Fluorat 02 (Lumex, Russia) fluorescence spectrophotometer.

Electrical measurements were performed with a pair of Ag/AgCl electrodes contacting the working buffer in compartments of the cell through salt bridges (micropipette plastic tips filled with agar and 0.1 M KCl). The electrical resistance of electrodes with salt bridges did not exceed 50 kΩ. Membrane capacitance and conductance were continuously measured as described by Sokolov and Pohl (2009). Triangular voltage waves were applied to the membrane, and the resulting current was recorded and analyzed with the aid of self-made software.

The change of membrane boundary potential, Δ*φ*_*b*_, after adsorption and damage of charged or dipole molecules at one side of the BLM was measured by the inner field compensation (IFC) technique (Sokolov and Kuz’min, 1980; Ermakov and Sokolov, 2003). DPhPC lipids differ by their hydrocarbon tails from the typical bacterial ones, however, they give much more stable membranes for long-time experiments, showing the same behavior in IFC experiments as membranes from bacterial and eukaryotic lipids (Batishchev et al., 2016). The boundary potential can be presented as a sum of the surface potential measured in the diffuse part of the electrical double layer and the dipole potential arising from the mutual orientation of water dipoles in proximity to the membrane and dipoles of polar heads of lipids. According to the Gouy-Chapman model (Gouy, 1910; Chapman, 1913), the surface potential of the lipid bilayer depends on its surface charge density and ionic strength of electrolyte solution and mainly reflects the adsorption of charged molecules at the interface between polar lipid headgroups and water solution. If adsorbing charged molecules can incorporate between lipid polar heads, it will change the dipole potential of the membrane. However, IFC alone cannot distinguish between changes of surface and dipole parts of the boundary potential to provide information about position of the molecule in the membrane. For independent determination of the potential drop in the diffuse part of the electrical double layer we studied electrophoretic mobility of liposomes by applying the method of dynamic light scattering using Zetasizer II (Malvern Instruments, United Kingdom) supplied with correlator PhotoCor SP (United States). Electrophoretic mobility spectrum was calculated using the software developed by the authors on the base of algorithm of the Malvern Company. Mobility spectrum generally had a complex shape and for the final analysis only peaks with maximal amplitude were used. The value of ζ-potential was calculated from mobility using the Smoluchowski equation. Liposomes were prepared from DPhPC. The lipid solution in chloroform was rotary evaporated under vacuum from a round bottom glass flask for approximately 50 min. Then the working buffer was added and shaked by BioVortex VI. The final concentration of lipids in the solution was 1 mg/mL.

The photodynamic efficiency of the phosphorus(V) porphyrin complexes at the BLM was evaluated by measuring the rate of oxidation of the molecules of di-4-ANEPPS used as targets of singlet oxygen. Solutions of the porphyrin and di-4-ANEPPS in ethanol were added together into the compartment far from the light source to prevent the attenuation of the light beam by its absorption in water. The membrane was illuminated by a semiconductor laser STAR405F10 (Roithner Laser Technik, Austria) with a wavelength of 405 nm. The optic power of illumination varied from 0.1 to 1 mW by passing the light through a grey filter. The rate of oxidation of the di-4-ANEPPS was determined by fitting the kinetics of the change of the boundary potential difference during the illumination phase and its following restoration in the dark phase:

$$R={\left.\frac{d\phi_{rel}\left(t\right)}{dt}\right|}_{t=0},or\;R=\frac{1}{\tau_{L}}-\frac{1}{\tau_{D}}$$ (1)

where $\phi_{rel}\left(t\right)=\frac{\phi \left(t\right)}{\phi_{ads}}$ ; *φ*_*ads*_ is the boundary potential difference arising due to adsorption of di-4-ANEPPS prior to the illumination, *φ(t)* is the potential difference measured after the beginning of the illumination, *τ*_*L*_ and *τ*_*D*_ are time constants of exponents approximating the kinetics of *φ(t)* during the illumination phase and the following dark phase, respectively.

### Quantum chemistry calculations and molecular dynamics simulations

All-atom molecular dynamics simulations of typical bacteria mixed 1,2-dioleoyl-sn-glycero-3-phosphocholine (DOPC)/1,2-dioleoyl-sn-glycero-3-phospho-(1′-rac-glycerol) (DOPG) (70/30 by moles) bilayer consisting of 128 lipids and 4 phosphorus(V) porphyrin molecules were performed to elucidate the interaction of the porphyrins with the lipid bilayer. All force-field parameters, except for phosphorus(V) porphyrin molecules, were taken from an all-atom CHARMM36 force field (Huang and MacKerell, 2013). We used a method based on the force field toolkit plugin (fftk), which was introduced by Pavlova et al. (2018) for the parametrization of corrinoids, to develop force field parameters that represented phosphorus(V) porphyrin molecules and were compatible with the CHARMM force field (Mayne et al., 2013).

Parameters for pyridyl and phenyl groups of phosphorus(V) porphyrin were taken from CGenFF (Vanommeslaeghe and MacKerell, 2012). Model compounds representing core rings were used to determine the main set of force field parameters (**n**(**OH)**_**2**_ and **n(OEt)**_**2**_ models). Additionally, we used a slightly larger system containing one phenyl group to determine linking parameters between coring rings and equatorial groups. Four different atom types for N were introduced to take into account the asymmetry of the porphyrin ring. Most of the dihedrals containing N1-N4 and P atoms were set to zero to reduce the risk of overfitting. Force field parameters were optimized based on the quantum mechanical data calculated with the ORCA program package (Neese, 2018). MP2 and the 6–31G* basis set were used for geometry optimization, potential energy scan, and hessian calculation. Molecular electrostatic potential (MEP) calculation and natural population analysis (NPA) (Reed et al., 1988) were performed with HF and the 6–31G* basis set. Partial charges were obtained by combining the restrained electrostatic potential (RESP) (Bayly et al., 1993) approach and NPA. NPA was used to determine charges for N1-N4 and P, remaining charges were calculated with the RESP method. Bonded parameters were optimized with fftk as described by Mayne et al. (2013). Lennard-Jones (LJ) parameters for all atoms except P were set by analogy from CGenFF, and LJ parameters for P were set to zero because it does not have an open site. Detailed information about obtained parameters and parametrization procedures are presented in the Supplementary Material section “Parametrization of the Force Field”.

Initially, two phosphorus(V) porphyrin molecules were placed inside the lipid membrane and two molecules were placed below and above the lipid membrane at a distance of approximately 1.5–2 nm. The system containing lipid membrane and porphyrin molecules was fully hydrated with ∼6600 TIP3P water molecules and neutralized with Na^+^ ions. The GROMACS 2019 (Van Der Spoel et al., 2005; Hess et al., 2008) package was used to perform the molecular dynamic simulation. A time step of 2 fs was used for integration. All bonds were constrained using the LINCS algorithm (Hess, 2008). During the equilibration, pressure and temperature were maintained constant with the Berendsent thermostat and barostat at 1 bar and 300 K, respectively, (Berendsen et al., 1984). For the production run, the thermostat was switched to the Noose-Hoover thermostat (Hoover, 1985) and the barostat to the Parinello-Rahman barostat (Parrinello and Rahman, 1981).

Electrostatics was treated using the particle mesh Ewald (PME) method (Darden et al., 1993; Essmann et al., 1995) with a cut-off of 1.2 nm. Lennard-Jones interactions were smoothly switched to zero from 1 to 1.2 nm. We used two temperature coupling groups: one for membrane and phosphorus(V) porphyrin molecules and the other for water and ions.

The following equilibration protocol was used. The initial system was minimized using the steepest descent algorithm with 5,000 steps, followed by a short 500 ps simulation in the NVT ensemble for heating up the system. To equilibrate the density, an additional round of 5 ns simulation in the NPT ensemble was performed. After the equilibration procedure, a 300–400 ns MD simulation in the NPT ensemble was carried out for all the systems. The position and orientation of porphyrin molecules were analyzed over the last 100 ns of the trajectory and only porphyrin molecules equilibrated inside the lipid bilayer at more than 100 ns were used.

### Experiments with bacteria

To test the antibacterial activity of PS molecules we used strains of Gram-negative bacteria *E. coli T61* and *A. baumannii NIH61*. All compounds were tested in a range from 0.39 μM to 100 μM in doubling dilutions. Ampicillin for *E. coli* and Colistin for *A. baumannii* were used as controls. Bacterial colonies were incubated in 5 mL of the LB media (Sigma-Aldrich, Saint-Louis, MO, United States) for 24 h at 37°С and 220 rpm. In total, 250 µL of the suspension was mixed with 4.75 mL of the LB media. Further, we added 200 μL of the suspension with appropriate dilutions of antimicrobial compounds to each well of a 96-well plate and incubated for 2 min. Then, wells were irradiated for 1 minute with a 405 nm laser (with a power of 100 mW, light dose 5 J/cm^2^) and incubated for 16 h at 37°С. The concentration that inhibited 50% of the bacterial growth (minimal inhibitory concentration, MIC_50_) was determined as the optical density at 600 nm (OD_600_) averaged from three independent experiments. To find bacterial colonies that survived at the two times higher concentration, 100 μL of the suspension from wells with a double MIC_50_ of the PS were serially diluted 10^5^–10^8^ times, transferred to 10 cm Petri dishes with the 1.5% agar medium (Sigma-Aldrich, Saint-Louis, MO, United States), and incubated for 16 h. Colonies were counted, and the percentage of survived colonies was estimated as the ratio of the number of colonies after irradiation to the control. The control group was colonies with irradiation without added phosphorus(V) porphyrins. The results were averaged from three independent experiments.

## Results and discussion

### Synthesis and photophysical properties of phosphorus(V) porphyrins

Free-base porphyrins **1-3** were prepared following the standard procedures (Adler et al., 1967; Barton et al., 2000). Phosphorus (V) complexes were synthesized using previously reported methods (Meshkov et al., 2016; 2017) (see Supplementary Figures S1–S4). The photophysical properties of the compounds from Chart 1 are presented in Table 1. The quantum yield of singlet oxygen generation in chloroform increased with the number of pyridyl groups for all of the studied phosphorus (V) porphyrins. For water and DMSO solutions such increase was detected for **n(OH)**_**2**_, while for **n(OEt)**_**2**_ Φ_Δ_ values in these solvents changed non-monotonously, with the maximal value for **2(OEt)**_**2**_.

**TABLE 1:** Photophysical properties of the phosphorus (V) porphyrins.

Compound	Chloroform		DMSO		Water	
	Φ_Δ_	Φ_f_	Φ_Δ_	Φ_f_	Φ_Δ_	Φ_f_
1(OH)_2_ ^a^	0.73	0.027	0.24	0.126	0.11	0.119
2(OH)_2_ ^a^	0.99	0.029	0.31	0.119	0.29	0.113
3(OH)_2_	1.00	0.010	0.37	0.110	0.34	0.070
1(OEt)_2_ ^a^	0.77	0.036	0.34	0.125	0.12	0.073
2(OEt)_2_ ^a^	0.99	0.060	0.37	0.104	0.46	0.083
3(OEt)_2_	1.00	0.030	0.21	0.100	0.30	0.70

^a^ From Meshkov et al. (2017).

### Adsorption of phosphorus (V) porphyrins at the bilayer lipid membrane (BLM)

We recently demonstrated that lipid membrane binding correlates with the photodynamic efficacy of porphyrins (Jiménez-Munguía et al., 2019). To analyze the adsorption of phosphorus(V) porphyrin complexes in the BLM we monitored the change of the boundary potential difference (Δ*φ*_*b*_) across the membrane using the IFC technique (Sokolov and Kuz’min, 1980). Despite bacteria having lipopolysaccharides in outer membranes, which were not included in our models, existing studies suggest that oxygen required for the photodynamic activity of PSs is accumulated mainly in the lipid bilayer of bacterial membranes (Maisch et al., 2007). The cationic nature of PSs allows them to bind lipopolysaccharides and further penetrate the lipid matrix of their membranes (Ball et al., 1999; Tim, 2015; Ghazal et al., 2017; Miretti et al., 2017). Therefore, in our studies, we focused on the binding and arrangement of PS molecules in the lipid bilayer.

**FIGURE 1:**
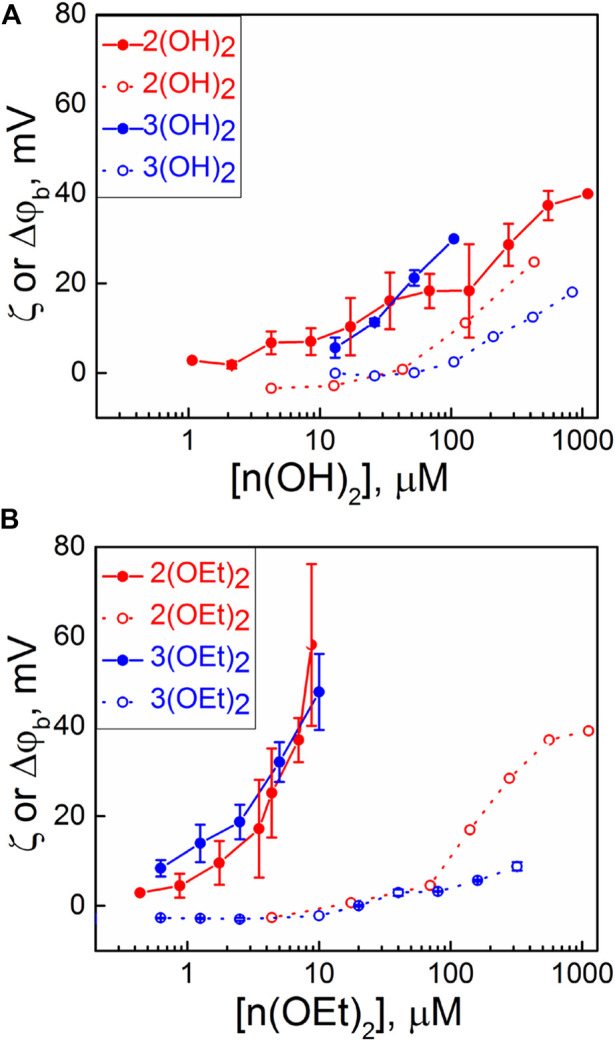
Dependence of Δ*φ*_*b*_ (IFC, filled symbols) on BLM and ζ-potentials of liposomes (open symbols) on the bulk concentration of compounds containing: **(A)** OH axial groups and **(B)** ethoxy axial groups. Error bars are standard deviations from 3–5 repetitions.

Each addition of a PS caused a change of the boundary potential difference, Δ*φ*_*b*_, at the membrane, which was monitored until a steady state was established. Dependencies of the steady-state values of Δ*φ*_*b*_ on the concentration of compounds in the solution are presented in Figure 1 (filled symbols). The addition of **1(OH)**_**2**_ and **1(OEt)**_**2**_, which have no pyridyl peripheral groups, did not cause the change of Δ*φ*_*b*_. Other phosphorus(V) porphyrin complexes showed the rise of Δ*φ*_*b*_ in a concentration-dependent manner. Values of Δ*φ*_b_ were higher for **n(OEt)**_**2**_ compounds compared to **n(OH)**_**2**_ ones. To distinguish between the surface and dipole part of the boundary potential, thus providing information about the penetration depth of the compounds, we measured the ζ-potential of liposomes with various concentrations of porphyrins in the solution. Usually, the values of the ζ-potential are close to the potential drop in the diffuse part of the electric double layer (Ermakov and Sokolov, 2003; Sokolov and Mirsky, 2004), which depends on the surface charge of the membrane and ionic strength of the solution. Values of the ζ-potential obtained for compounds presented in Chart 1 are plotted in Figure 1 (open symbols). The slopes of the dependences of the ζ-potential on the concentration of porphyrin in water were higher for porphyrins with one pyridyl group than that with two groups. This can be explained by the different charges of these molecules (Sokolov and Mirsky, 2004). The values of the ζ-potentials did not differ significantly between porphyrins with ethoxy and OH groups. This means that the surface charge and, hence, the amount of the porphyrin molecules at the interface between the membrane and the water solution was almost the same for both types of porphyrins. In total, the differences between values of Δ*φ*_*b*_ and ζ-potential were much bigger for **n(OEt)**_**2**_ compounds compared to **n(OH)**_**2**_ ones. Generally, the discrepancy between these potentials indicates either the immersion of the charged groups of the molecules into the membrane or the presence of oriented dipoles of these molecules. This allows concluding that it is the nature of the axial ligand and not the amount of phenyl and pyridyl groups that significantly influences the penetration depth of porphyrins into the BLM. 

It is noteworthy that compounds **1(OH)**_**2**_ and **1(OEt)**_**2**_, which have only phenyl substituent, did not show any change in both ζ-potential and Δ*φ*_*b*_. However, these substances adsorb at the membrane, as it will be shown below in studying their photodynamic efficiency in the BLM.

### The photodynamic efficiency of the porphyrins in the BLM

**FIGURE 2:**
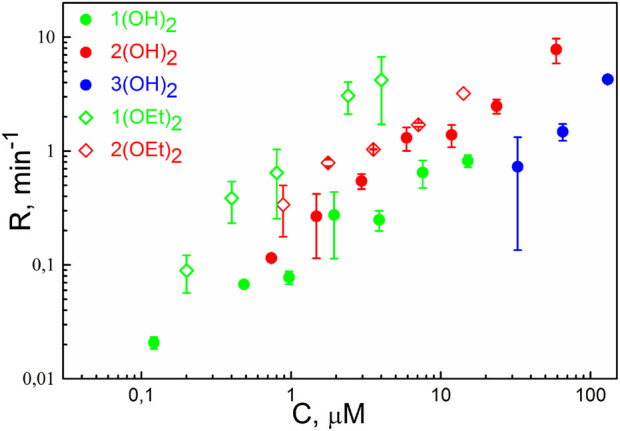
The rate of oxidation of di-4-ANEPPS as a function of concentration (C) of phosphorus(V) porphyrins in the solution. Irradiation was performed at λ = 405 nm. Error bars are standard deviations from 3-5 repetitions.

The photodynamic efficiency of the porphyrins was studied similarly to an earlier reported procedure (Sokolov et al., 2016). In brief, we measured the oxidation rate, *R*, of the styryl dye di-4-ANEPPS, which served as a target molecule for singlet oxygen. The dependence of *R* on the concentration of porphyrins in the solution is presented in Figure 2. We observed the oxidation of the target molecule even with the porphyrins **1(OH)**_**2**_ and **1(OEt)**_**2,**_ meaning that these molecules do adsorb at the membrane. The dependence of *R* on the concentration for **1(OEt)**_**2**_ was above others for the whole range of concentrations, and much higher than for **1(OH)**_**2**_. Values of *R* for **3(OH)**_**2**_, which also demonstrated the membrane perturbation (see Figure 1A), were considerably less than all the other ones. Thus, solely the ability of the PS molecule to change the lipid packing is not enough to draw a conclusion about its photodynamic activity.

Illumination of the BLM with adsorbed porphyrins **2(OEt)**_**2**,_ **3(OH)**_**2**_, and **3(OEt)**_**2**_ led to a drop of the Δ*φ*_*b*_. This indicates that these compounds can be damaged under illumination, perhaps, due to oxidation by SO generated by the excited porphyrin molecules. Photobleaching of porphyrin molecules can decrease their number at the membrane and total yield of singlet oxygen, resulting in an underestimation of the value of *R*. To attenuate this effect, we decreased the intensity of the light 10 times, thus reducing the loss of porphyrin molecules at the membrane due to their self-damage. We normalized the result to the standard intensity using the earlier observation that the value of *R* is proportional to the intensity of light (Sokolov et al., 2016). It allowed evaluation of the oxidation rate *R* as a function of concentration for **2(OEt)**_**2**_ and **3(OH)**_**2**_. However, we failed to evaluate *R* for **3(OEt)**_**2**_, where the change of the Δ*φ*_*b*_ due to the self-damage of the porphyrin was comparable with that resulted from the oxidation of di-4-ANEPPS. In general, these results correlated with the previous studies of the photostability of porphyrin molecules: the introduction of pyridyl groups into tetraphenyl porphyrin molecules decreases its photostability (Meshkov et al., 2017).

To compare the behavior of phosphorus(V) porphyrins with the photosensitizer with the established antibacterial activity, Methylene Blue, we performed the same measurements for this molecule. The addition of Methylene Blue to the membrane was not followed by a change of the boundary potential difference at the BLM. Nevertheless, the illumination of the BLM after adsorption of di-4-ANEPPS, either at the same side of the membrane (*cis*) or at the opposite side (*trans*) with Methylene Blue, caused the damage of the target molecule registered as a drop of Δ*φ*_*b*_ (see Supplementary Figure S5). This result correlated with the membrane binding activity of **1(OH)**_**2**_ and **1(OEt)**_**2**_, which also did not show a change in Δ*φ*_*b*_, and demonstrated photodynamic efficiency at the BLM.

### Molecular dynamics simulations of phosphorus(V) porphyrin adsorption at the BLM

To characterize the interaction of phosphorus(V) porphyrins with the lipid membrane at the atomic level we performed all-atom molecular dynamics simulations. Force field parameters were developed based on the quantum chemical calculations, as described in the Supplementary Material, section “Parametrization of the Force Field”. We monitored the adsorption of porphyrins by analyzing the temporal evolution of their center of mass. Time scales of the porphyrin membrane binding observed in the simulations ranged from 50 ns to 250 ns and had a positive correlation with the number of pyridyl groups (Supplementary Figure S6). Only one molecule of **3(OEt)**_**2**_ did not enter the lipid membrane interior during the simulation. After membrane binding, phosphorus(V) porphyrin molecules occupied a position below the region of lipid polar headgroups and remained there for the rest of the simulation. We also observed relocation to the water-lipid interface of all porphyrin molecules that were initially placed at the center of the lipid membrane. In all cases, these molecules moved to the region of lipid headgroups in less than 50 ns. This indicates a relatively large energy barrier for the membrane translocation event. After binding and migration events, it took approximately 100 ns for phosphorus(V) porphyrin molecules to reach their equilibrium position and orientation.

**FIGURE 3:**
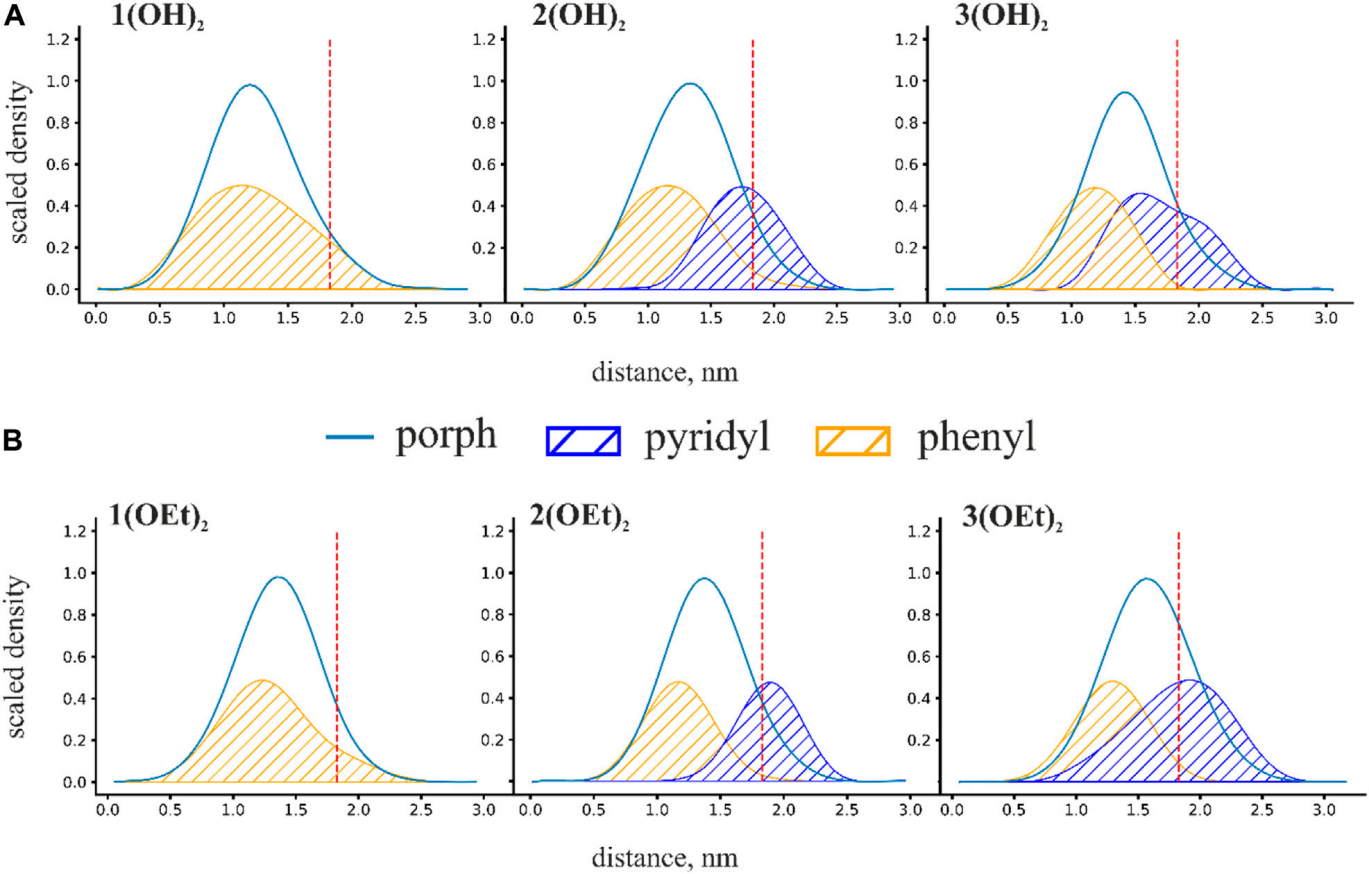
Scaled mass density profiles for **(A) n(OH)**_**2**_ and **(B) n(OEt)**_**2**_ porphyrins. The red dashed line in these figures represents the phosphate group of lipids and is defined here as the water-lipid interface. Distance is calculated from the intermonolayer surface of the bilayer lipid membrane.

To obtain insight into the spatial arrangement of porphyrins inside the lipid membrane we calculated mass density profiles of phosphorus(V) porphyrins and their equatorial groups. Scaled mass density profiles of **n(OH)**_**2**_ and **n(OEt)**_**2**_ porphyrins are presented in Figure 3. The red dashed line represents the phosphate group of lipids and is defined here as the water-lipid interface. Obtained mass density profiles were very wide, covering almost the whole interior of the membrane. The width of density profiles was similar for all porphyrins, but the position of the mass density distribution varied with the presence of pyridyl groups: an increase in the number of pyridyl substituents shifted the molecule from the center of the membrane to the water-lipid interface. For **n(OH)**_**2,**_ we observed a broader range of positions of both phenyl and pyridyl groups (Figure 3A) compared to **n(OEt)**_**2**_ ones (Figure 3B), meaning more active movements of **n(OH)**_**2**_ porphyrins inside the membrane. The maximum pyridyl density was located close to the membrane surface, indicating that pyridyls prefer to be partially hydrated. The position of this peak at the region of lipid phosphate moieties reflects the disturbance of the lipid packing by phosphorus(V) porphyrin molecules. Thus, this effect should be the most pronounced for **2(OEt)**_**2**_ and **3(OEt)**_**2**_ porphyrins, and less for **3(OH)**_**2**_, which has only one shoulder of the mass density distribution of pyridyl groups to the right of the lipid phosphate position (see Figure 3). For the **3(OH)**_**2,**_ this disturbance should be minimal. These observations are in line with the IFC and ζ-potential measurements (Figure 1), which indicated the increase in discrepancy between boundary potentials and ζ-potentials for **n(OEt)**_**2**_ compounds compared to **n(OH)**_**2**_ ones.

We characterized the penetration depth of porphyrins as a position of the maximum of the mass density profiles. The penetration depth for porphyrins is strongly linked with the degree of hydrophobicity. In the case of **n(OEt)**_**2**_ porphyrins, the position of the maximum of the mass density profiles shifted from ∼1.3 nm for **1(OEt)**_**2**_ to ∼1.5 nm for **3(OEt)**_**2**_ (Figure 3B). A similar shift was observed for porphyrins with OH axial groups: from ∼1.2 nm for **1(OH)**_**2**_ to ∼1.4 nm for **3(OH)**_**2**_ (Figure 3A). Surprisingly, the replacement of the OH group with a more hydrophobic ethoxy group resulted in a decrease in the penetration depth of the phosphorus(V) porphyrin molecule. This indicates that a simple increase in hydrophobicity has a limited effect on the penetration depth and that other factors, such as the orientation of the whole molecule in the lipid bilayer, should be taken into account.

**FIGURE 4:**
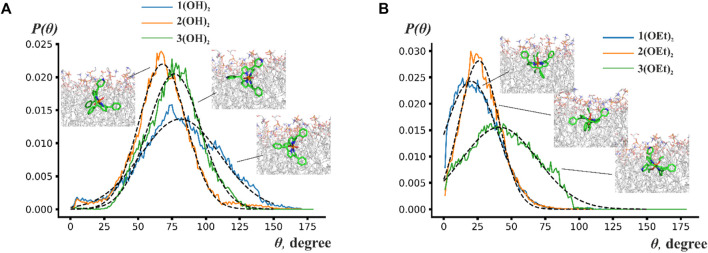
Probability distribution P(Θ) of the angles between the normal to the porphyrin plane and to the membrane plane for **(A)** **n(OH)**_**2**_ porphyrins and **(B) n(OEt)**_**2**_ porphyrins. Insets show snapshots from molecular dynamics simulations indicating the most probable position of the phosphorus(V) porphyrin molecules.

To analyze the orientation of phosphorus(V) porphyrin molecules inside the lipid membrane we calculated the normalized probability distribution P(Θ), where Θ is the angle between the normal to the porphyrin plane and the *z*-axis, which was perpendicular to the membrane plane. For a more quantitative description, the Gaussian function was used to approximate this distribution. Obtained probability distributions and snapshots of the most probable orientations are presented in Figure 4.

Figure 4 shows that porphyrin rings rotate by ∼70°–150° around some average value. This wide range of rotation implies that porphyrins are orientationally flexible inside the lipid bilayer. The central ring of **n(OH)**_**2**_ porphyrins preferred to be perpendicular to the membrane plane with maxima of Θ at ∼78° for **1(OH)**_**2**_ and **3(OH)**_**2**_, and ∼57° for **2(OH)**_**2**_. Contrary to **n(OH)**_**2**_ porphyrins, **1(OEt)**_**2**_ and **2(OEt)**_**2**_ preferred to stay parallel to the membrane surface with an average angle of ∼20° and ∼26°, respectively. The central ring of **3(OEt)**_**2**_ was oriented at ∼41° to the membrane plane. Figure 4A shows that **2(OH)**_**2**_ and **3(OH)**_**2**_ have similar ranges of rotation, while the range of rotation of **1(OH)**_**2**_ is considerably larger. Previously, it has been suggested that a reduction of the rigidity of the orientation leads to a higher energy loss of the excited state (Wainwright, 1998). It could explain the decrease of photodynamic efficiency for **1(OH)**_**2**_ compared to **2(OH)**_**2**_ (see Figure 2) despite the deeper insertion of **1(OH)**_**2**_ into the lipid membrane.

Therefore, only porphyrins **1(OEt)**_**2**_ and **2(OEt)**_**2**_, which demonstrated the highest oxidation rate of the target molecule in the BLM, stayed parallel to the membrane plane. The difference in orientation for **n(OH)**_**2**_ and **n(OEt)**_**2**_ indicated that the ethoxy group preferred to align along acyl chains of lipids. Overall, porphyrins were positioned in a way that their ethoxy groups were parallel to the *z*-axis, pyridyl groups were attached to the polar part of the membrane, and phenyl groups stayed as deep as possible into the hydrophobic part of the membrane. These observations are in line with the study by Cordeiro et al. (2012) and obtained difference between Δ*φ*_*b*_ and ζ-potential values for **2(OEt)**_**2**_ (Figure 1B), indicating high perturbance of the membrane in the region of lipid headgroups. Even though we were not able to detect the effect of compound **1(OEt)**_**2**_ on membrane structure from the IFC and ζ-potential measurements, MD simulations demonstrated that it stayed deeper into the membrane interior compared to **2(OEt)**_**2**_. Other phosphorus(V) porphyrins lay almost perpendicular to the membrane surface.

### Analysis of the antibacterial activity of phosphorus(V) porphyrins

**FIGURE 5:**
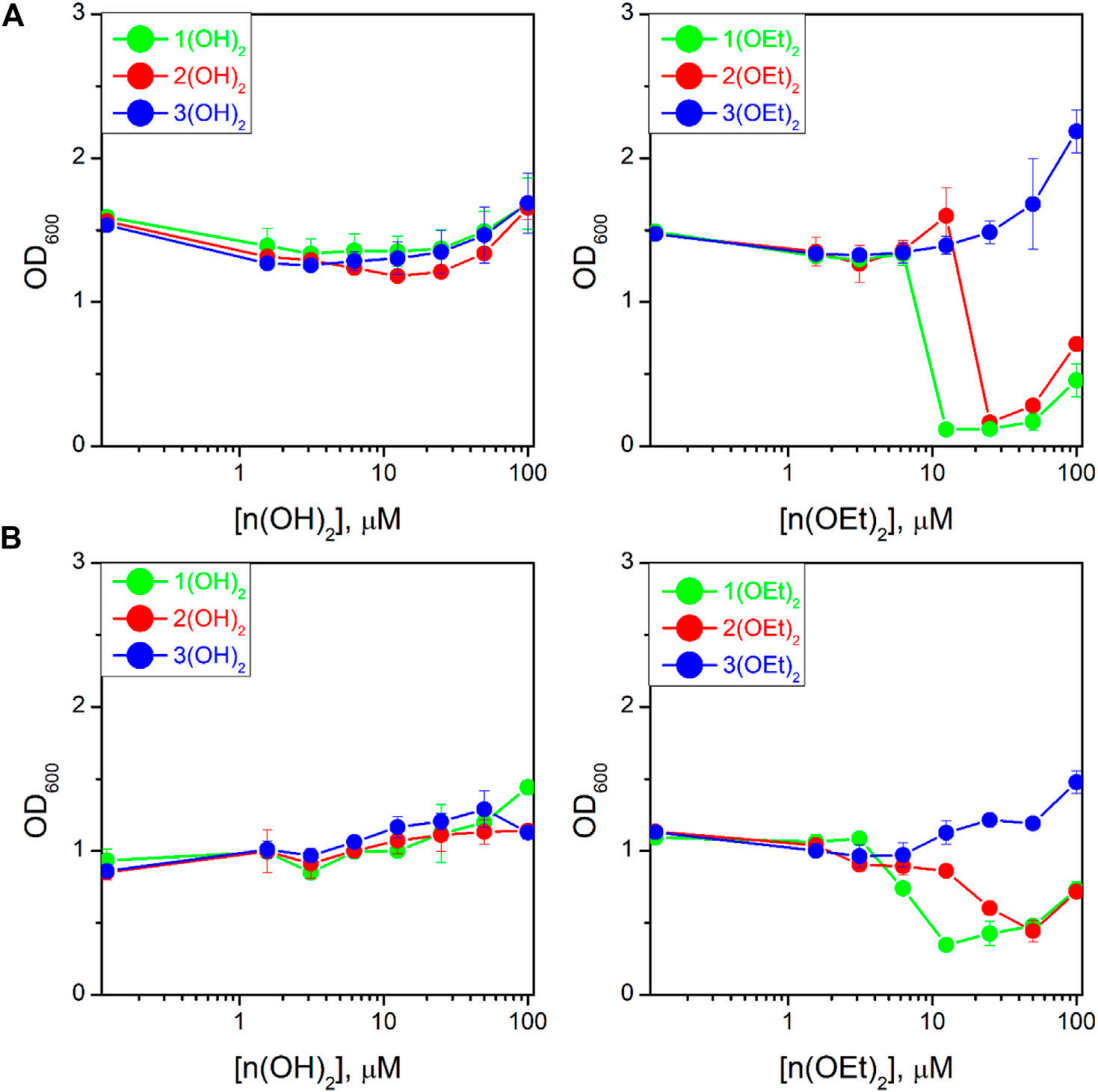
Dependence of the optical density at 600 nm (OD_600_) for the bacterial suspension of *Escherichia coli* **(A)** and *Acinetobacter baumannii* **(B)** on PS concentration after 16 h of incubation upon irradiation. The study was carried out in three independent repetitions and the error was determined by ANOVA.

We studied the antibacterial activity of six compounds presented in Chart 1 on the T61 strain of *E. coli* and the NIH601 strain of *A. baumannii*. Our goal was to make a photosensitizer with the highest efficacy after minimal incubation and irradiation times, therefore, we fixed the incubation time before irradiation to 2 min, and the light dose to 5 J/cm^2^. These parameters were not varied. Figure 5 shows the optical density at 600 nm (OD_600_) for *E. coli* (Figure 5A) and *A. baumannii* (Figure 5B) treated with phosphorus(V) porphyrins in such experimental conditions. We found that porphyrins containing a hydroxyl group as axial ligands had no effect on both bacteria (Figure 5, left column).

Compounds with ethoxy axial ligands exhibited cytotoxic activity depending on peripheral groups (Figure 5, right column): they decreased in the order of **1(OEt)**_**2**_-**2(OEt)**_**2**_-**3(OEt)**_**2**_, with the latter one even increasing bacteria growth compared to the control. Although the treatment of the cells with **1(OEt)**_**2**_ and **2(OEt)**_**2**_ demonstrated the same level of OD_600_, we observed that incubation with **1(OEt)**_**2**_ had a lower MIC_50_ on both cell lines (6.2 ± 0.5 μM (5.0 ± 0.4 μg/mL) for *E. coli* and 6 ± 1 μM (4.9 ± 0.8 μg/mL) for *A. baumannii*) than **2(OEt)**_**2**_ (39 ± 4 μM (32 ± 3 μg/mL) for *E. coli* and 18 ± 1 μM (15 ± 1 μg/mL) for *A. baumannii*). An increase of OD_600_ values at 100 µM of the tested compound might be associated with the absorbance of light by PS molecules in the solution (Figure 5). As a control, we used Ampicillin for *E. coli* and Colicin for *A. baumannii* (Supplementary Figure S12) with 24 h incubation. They demonstrated MIC_50_ values as 11 ± 1 μg/mL and 6 ± 1 μg/mL, respectively. Thus, MIC_50_ values for **1(OEt)**_**2**_ were the same as for *A. baumannii* incubated with Colicin, and even two times better than for *E. coli* treated with Ampicillin. To thoroughly analyze the activity of photosensitizers, we took 100 µL of the suspension from wells with a concentration of 12.5 µM (approximately two times higher than MIC_50_) for both bacterial lines after incubation with **1(OEt)**_**2**_ overnight. As a result of incubation, we calculated the relative (to the control) number of colonies that survived at this concentration. For the given dilutions (see Materials and Methods), we did not detect any colony of *A. baumannii* after the treatment with **1(OEt)**_**2**_, while for *E. coli* 2.2% ± 1.7% of the colonies survived. Thus, in the case of *A. baumannii,* a concentration of 12.5 µM can be interpreted as the minimal bactericidal concentration (MBC), and this concentration is also close to the MBC of *E. coli*.

## Conclusion

Antibacterial photodynamic therapy (aPDT) is believed to be a promising alternative to classic antibiotics. In principle, PS molecules can be attenuated for different wavelengths of light by choosing appropriate axial ligands and peripheral groups, thus allowing even intravenous application and further deep irradiation. However, the development of aPDT requires simultaneous solving of many tasks, such as selectivity towards bacteria, the photostability of PS, good cell binding and/or penetration, the ability to kill different bacterial strains, minimization of possible side effects, and even production costs. Therefore, we need good predictive tools and model systems for quick evaluation of the efficacy of PS molecules for aPDT. Several studies suggest that besides the high quantum yield of singlet oxygen, the lipid membrane affinity is a necessary prerequisite for the antibacterial activity of PS molecules (Engelmann et al., 2007b; Ethirajan et al., 2011; Jiménez-Munguía et al., 2019). However, the penetration depth and orientation of the photosensitizer into the lipid environment also regulate its photodynamic efficacy (Engelmann et al., 2007a; Cordeiro et al., 2012; Sokolov et al., 2018). Experimental investigation of all of these parameters is a very complicated task, thus, molecular modeling, such as MD simulations, is suggested as a promising tool to assess the lipid membrane interactions of PS molecules (Cordeiro et al., 2012). Simulations provide molecular insights into physicochemical mechanisms of PS-membrane interactions, thus, allowing us to discover structure-function relationships. Nevertheless, the MD approach should be accompanied by studying the model lipid membranes, which would allow for estimating the photodynamic efficacy of the PS.

Here, we presented a step-by-step approach based on biophysical studies on model membranes and MD simulations, which allowed finding a structure-activity relationship for three series of phosphorus(V) porphyrins with different peripheral groups and axial ligands. By measuring membrane potentials, we can draw conclusions about i) the ability of the PS molecule to disturb lipid packing, thus, immersing itself deep into the membrane, and ii) the photodynamic efficacy of the PS in the lipid bilayer, modeling bacterial cell membranes. Molecular dynamics simulations allowed us to find structural features of the PS molecule that are responsible for its maximal antibacterial activity. Thus, combining these approaches, we have, on one hand, a tool for the prediction of the molecular structure, and, on the other hand, a versatile technique for quick estimation of the photodynamic activity. In the present study, we utilized this combined approach for the set of phosphorus(V) porphyrins (see Chart 1). Among them, only **1(OEt)**_**2**_ and **2(OEt)**_**2**_ molecules showed beneficial antimicrobial activity against Gram-negative bacteria *E. coli* and *A. baumannii*, possessing lipopolysaccharide walls. As we demonstrated by MD simulations, these molecules stayed deep inside the membrane almost parallel to its surface. Compound **1(OEt)**_**2**_ demonstrated the highest aPDT activity (see Figure 5) and photodynamic efficacy in the BLM (see Figure 2). Other phosphorus(V) porphyrins, having even better quantum yield in both organic and water solutions, and immersing deep into the lipid bilayer, did not manifest antimicrobial activity. We demonstrated that these molecules lay almost perpendicular to the membrane surface. It was noteworthy that the quantum yield of singlet oxygen for both series of phosphorus(V) porphyrins increased with a change of phenyl groups to pyridyl ones, as measured in water, chloroform, and DMSO (see Table 1). Thus, the quantum yield of singlet oxygen in a solution does not clearly reflect the antibacterial activity of phosphorus(V) porphyrins.

Thus, our data indicate that the presence of ethoxy axial ligands increases the antibacterial activity of phosphorus(V) porphyrins in comparison with the hydroxyl groups, while the presence of a pyridyl substituent in the equatorial position of porphyrin decreases the antibacterial activity of phosphorus(V) porphyrin. As we demonstrated by the measurements of membrane potentials (see Figure 1), **n(OEt)**_**2**_ compounds have higher photodynamic efficacy in the BLM compared to **n(OH)**_**2**_ ones. Therefore, both penetration depth and orientation of the phosphorus(V) porphyrin molecules in the lipid bilayer regulate their applicability for aPDT. Our findings of a correlation between the structure of the PS molecule, its membrane arrangement, and antimicrobial efficacy manifested that lipid membrane model systems provide the most suitable and convenient environment to assess the antibacterial activity of different PS molecules. Using this approach, we found a new PS, Diethoxido (5,10,15,20-tetraphenylporphyrinato)phoshоrus(V) bromide, whose antimicrobial activity against *A. baumannii* was the same as for common antibiotic Colicin, and even better than for Ampicillin in case of *E. coli*. Our compound has similar MIC_50_ and MBC to other antimicrobial photosensitizers (Gamelas et al., 2022) but provides them after an extremely low light dose of 5 J/cm^2^. Moreover, our approach of a combination of biophysical experiments with BLM and MD simulations gives us a powerful tool for determining the structure-activity relationship, as well as a quick and reliable assessment of the photodynamic efficacy of PS molecules.

## Data Availability

The original contributions presented in the study are included in the article/Supplementary Material, further inquiries can be directed to the corresponding authors.
