# Supplementary material for: Antimicrobial activity of photosensitizers: arrangement in bacterial membrane matters
Source: Front Mol Biosci. 2023 May 15;10:1192794. doi: 10.3389/fmolb.2023.1192794 (PMC10226669; doi:10.3389/fmolb.2023.1192794)
Supplement: Supplementary file 1 [file DataSheet1.pdf]

SUPPORTING INFORMATION

Antimicrobial Activity of Photosensitizers: Arrangement in Bacterial Membrane Matters

Oleg V. Batishchev[†]*, Maksim A. Kalutskii[†], Ekaterina A. Varlamova[†], Anna N. Konstantinova[†], Kirill I. Makrinsky[†], Yury A. Ermakov[†], Ivan N. Meshkov[†], Valerij S. Sokolov[†], Yulia G. Gorbunova[†,‡]*.

† Frumkin Institute of Physical Chemistry and Electrochemistry, Russian Academy of Sciences, 31/4 Leninskiy pr. 119071 Moscow, Russia.

‡ Kurnakov Institute of General and Inorganic Chemistry, Russian Academy of Sciences, 31 Leninskiy pr. 119991 Moscow, Russia.

TABLE OF CONTENTS

Figure S1. ^{1}H-NMR (bottom, $CDCl_3$+CD_3OD, 600 MHz, 25 °C) and ^{31}P-NMR (top right, $CDCl_3$+CD_3OD, 243 MHz, 25 °C) spectra of **3(OH)₂** 2

Figure S2. HR-ESI MS spectrum of $3(OH)_2$........ 3

Figure S3. ^{1}H-NMR (bottom, CD_3OD, 600 MHz, 25 °C) and ^{31}P-NMR (top right, CD_3OD, 121 MHz, 25 °C) spectra of **$3(OEt)_2$**. 3

Figure S4. HR-ESI MS spectrum of **$3(OEt)_2$**. 4

Figure S5. The rate *R* of oxidation of di-4-ANEPPS adsorbed on BLM either at cis (filled symbols) or trans (closed symbols) side of the membrane with the Methylene Blue under illumination by the semiconductor laser 670 nm, 1 mW, as a function of the concentration of Methylene blue in the solution. 4

Figure S6. Trajectory of z coordinate of the center of mass of the porphyrin ring for studied porphyrins. **a**, **b** and **c** correspond to the **$1(OH)_2$-$3(OH)_2$** porphyrins, while **d**, **e** and **f** correspond to **$1(OEt)_2$-$3(OEt)_2$** ones. The average position of the phosphate group of lipid molecules is shown as a black dash line. 5

PARAMETRIZATION OF THE FORCE FIELD 6

Lennard-Jones (LJ) parameters assignment 6

Figure S7. Atom numbers used in this work. Colors illustrate the bonds around which the dihedral scans were performed. 6

Table S1. LJ parameters for heavy atoms. 6

Partial charges 7

Table S2. Partial charges determined with NPA. 7

Table S3. Partial charges of heave atoms determined with RESP. 7
Bond and angle parameters 7
Dihedral parameters 8
Table S4. RMSE for fitted dihedrals. 8
Figure S8. Comparison of QM and MM PESs for ring dihedrals. 9
Figure S9. Comparison of QM and MM PESs for ethoxy dihedrals. 10
Figure S10. Comparison of QM and MM PESs for phenyl dihedrals. 10
Comparison of QM and MM optimized geometry 10
Figure S11. Comparison of QM (red) and MM (blue) optimized geometry. 11
Figure S12. Dependence of the optical density at 600 nm (OD_{600}) for the bacterial suspension of *E. coli* incubated for 24 h with Ampicillin (**A**) and of *A. Baumannii* incubated for 24 h with Collicin (**B**). The study was carried out in 3 independent repetitions, the error was determined by ANOVA. 11
REFERENCES... 11

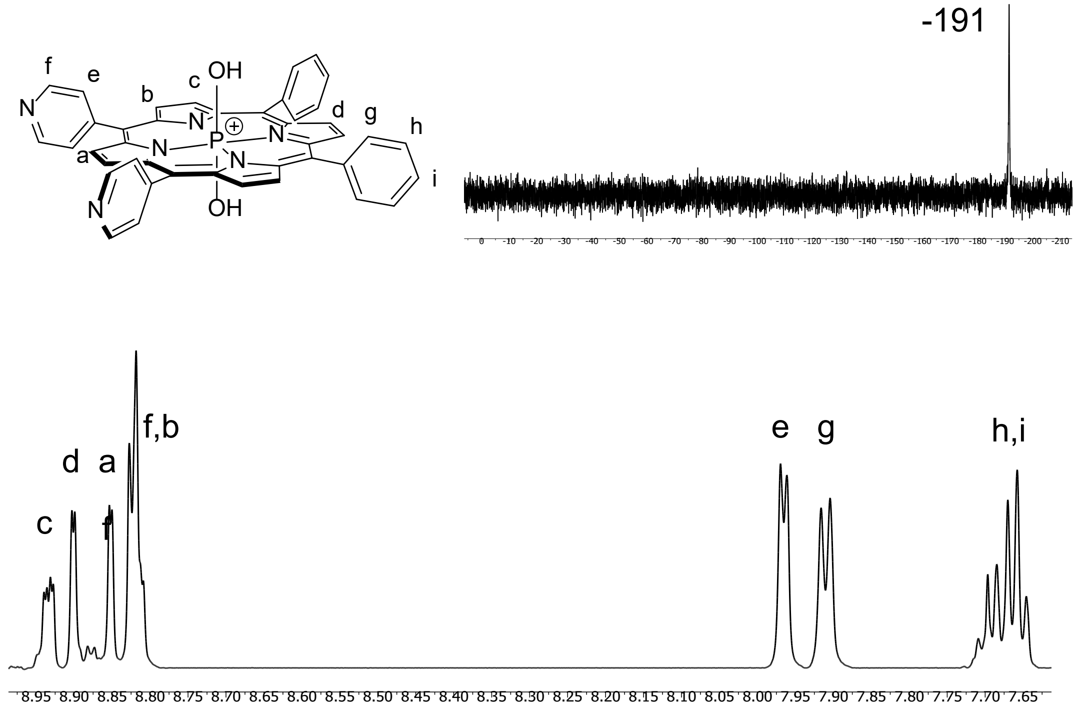

Figure S1. ^{1}H-NMR (bottom, $CDCl_3$+CD_3OD, 600 MHz, 25 °C) and ^{31}P-NMR (top right, $CDCl_3$+CD_3OD, 243 MHz, 25 °C) spectra of **3(OH)$_2$**

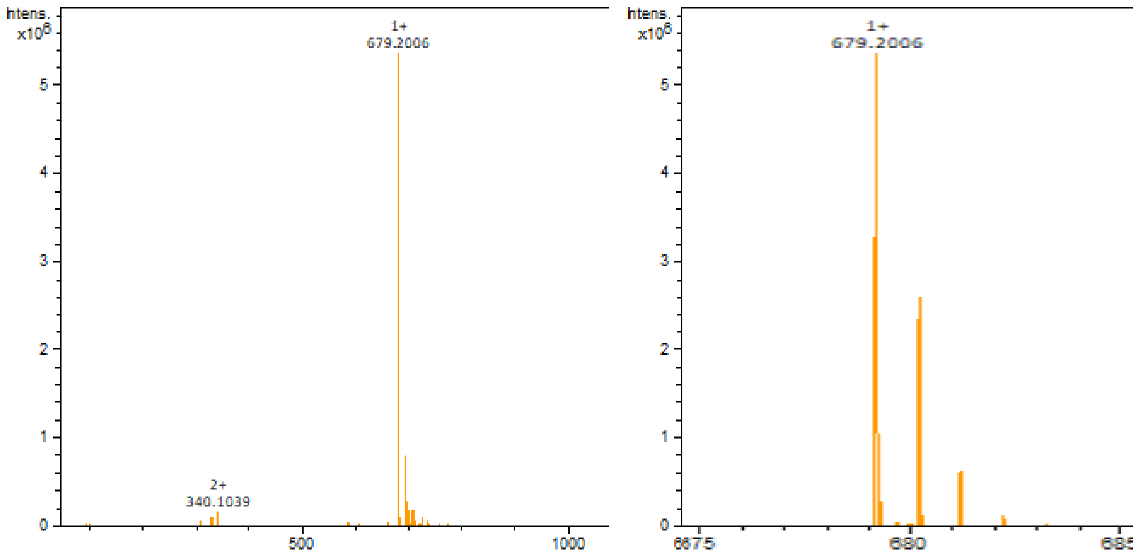

Figure S2. HR-ESI MS spectrum of 3(OH)2.

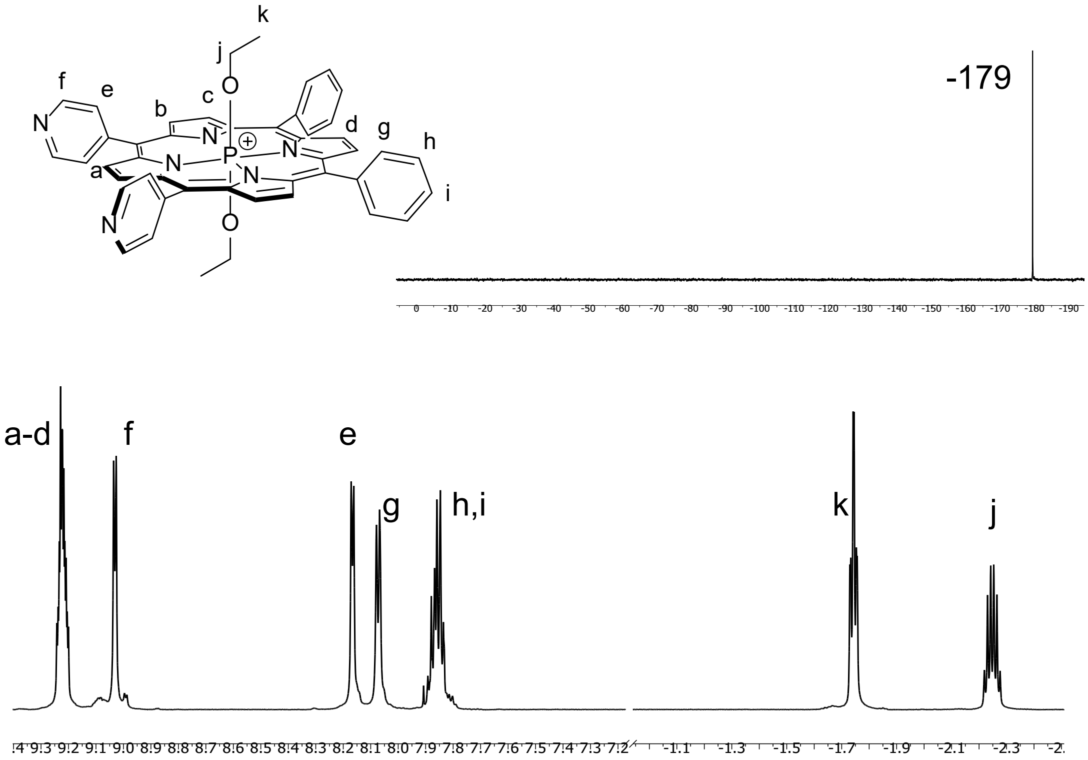

Figure S3. ^{1}H-NMR (bottom, CD$_3$OD, 600 MHz, 25 °C) and ^{31}P-NMR (top right, CD$_3$OD, 121 MHz, 25 °C) spectra of **3(OEt)$_2$**.

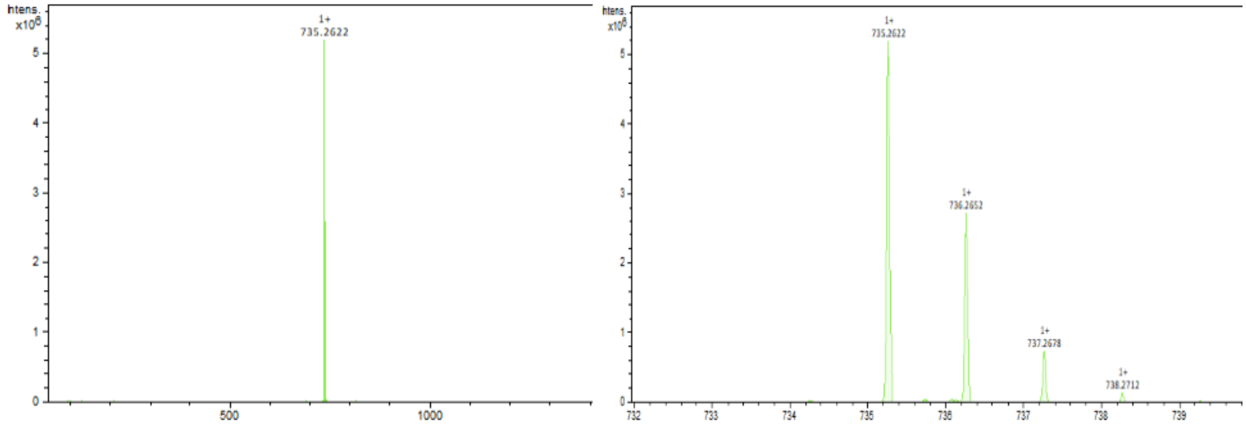

Figure S4. HR-ESI MS spectrum of **3(OEt)$_2$**.

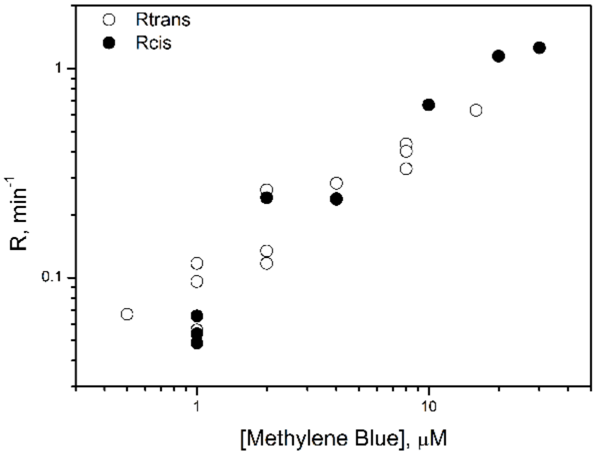

Figure S5. The rate *R* of oxidation of di-4-ANEPPS adsorbed on BLM either at cis (filled symbols) or trans (closed symbols) side of the membrane with the Methylene Blue under illumination by the semiconductor laser 670 nm, 1 mW, as a function of the concentration of Methylene blue in the solution.

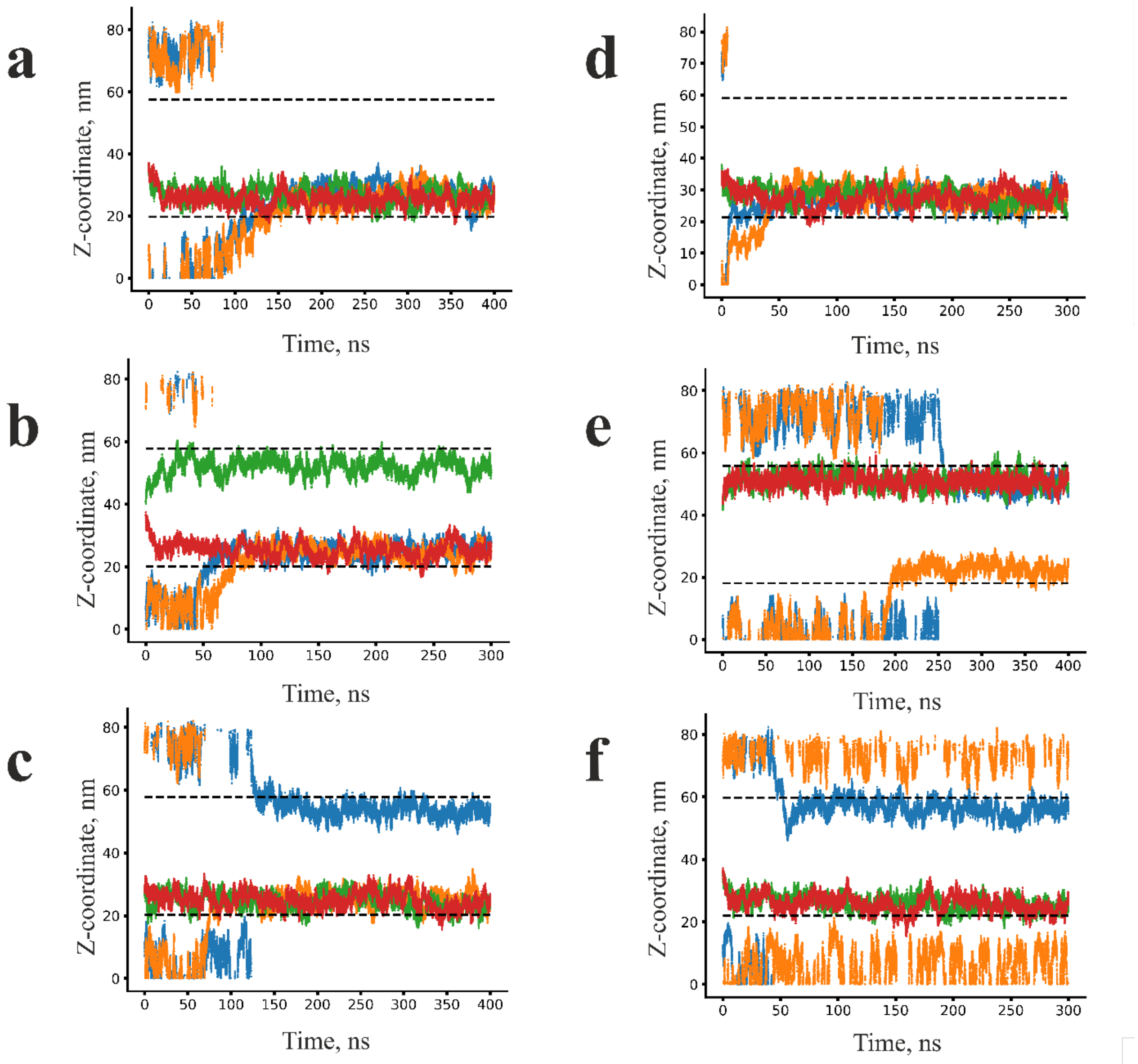

Figure S6. Trajectory of z coordinate of the center of mass of the porphyrin ring for studied porphyrins. **a**, **b** and **c** correspond to the **1(OH)$_2$-3(OH)$_2$** porphyrins, while **d**, **e** and **f** correspond to **1(OEt)$_2$-3(OEt)$_2$** ones. Each curve reflects the independent run of MD simulations. The average position of the phosphate group of lipid molecules is shown as a black dash line.

PARAMETRIZATION OF THE FORCE FIELD

Lennard-Jones (LJ) parameters assignment

Table S1 provide information about LJ parameter assignment. Figure S7 shows atom numbers. For hydrogen atoms connected to C1, C2, C5, C6, C10, C11, C15, C16 we used atom type HGR51, and for hydrogen atoms connected to C9, C14, C19, C20 we used aromatic hydrogen atom type HGR61. For hydrogen atoms connected to the oxygen, we used HGP1 atom type. For ethoxy group we used CG321 and CG331 for carbon atoms and HGA2 and HGA3 for hydrogen atoms.

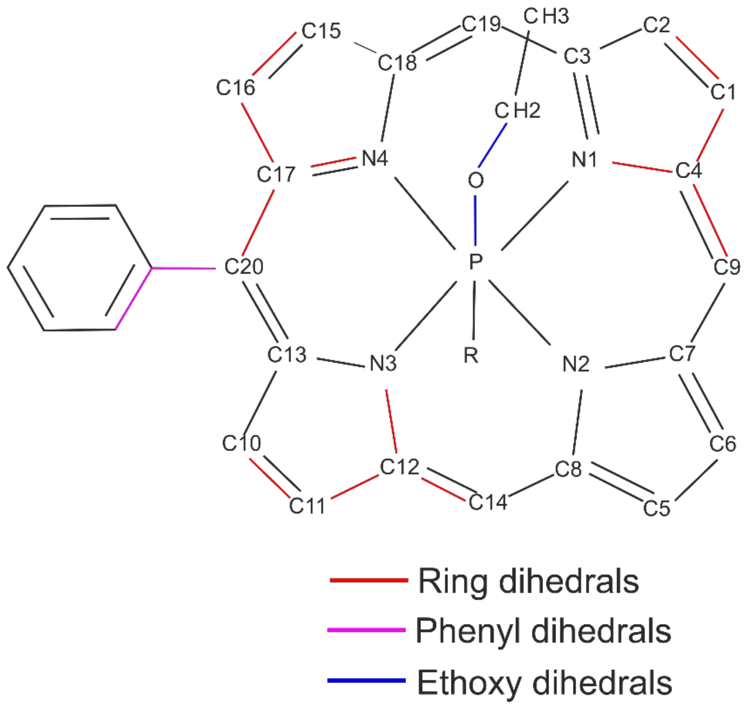

Figure S7. Atom numbers used in this work. Colors illustrate the bonds around which the dihedral scans were performed.

Table S1. LJ parameters for heavy atoms.

Atom number	CGenFF atom type	Atom types used in this work
C9, C14, C19, C20	CG2R61	CP
C3, C4, C7 C8, C12, C13, C17, C18	CG2R52	CPB
C1, C2, C5, C6, C10, C11, C15, C16	CG2R51	CPA
N1-N4	NG2R50	NP1-NP4
P	-	PG0
O1, O2	OG311, OG301	OG311, OG301

Partial charges

Partial charges for N and P atoms determined using NPA are provided in Table S2, and partial charges of heave atoms determined with RESP method with restrained charges for N and P are presented in Table S3. Charges for aromatic and aliphatic hydrogen atoms were assigned their standard value of 0.15 and 0.09 from CHARMM force field.

Table S2. Partial charges determined with NPA.

Atom	OH	OEt
P	2.662	2.688
N1	-0.766	-0.753
N2	-0.752	-0.74
N3	-0.735	-0.723
N4	-0.752	-0.74

Table S3. Partial charges of heave atoms determined with RESP.

Atom	OH	OEt
C1, C2, C5, C6, C10, C11, C15, C16	-0.242	-0.215
C3, C4, C7 C8, C12, C13, C17, C18	0.396	0.325
C9, C14, C19, C20	-0.32	-0.252
O1, O2	-0.8325	-0.609
C26, C27	-	-0.013
C28, C25	-	-0.254

Bond and angle parameters

We tested 3 sets of parameters based on the atom type assignment for nitrogen atoms: one atom type for all nitrogen atoms, three atom types based on obtained partial charges and four different atom types. Only parameter set with four different atom types for nitrogen atoms was able to correctly reproduce the optimized QM geometry (deviation less than 0.02 Å and 3° for bonds and angles, respectively).

Bonds and angles were fitted simultaneously to minimize the objective function described in ref.[1] with simplex optimization algorithm. We performed several runs of optimization until the objective function stopped decreasing. **n(OH)$_2$** model was used to obtain most of the bonded parameters for porphyrin ring. For **n(OEt)$_2$** model only bonded parameters connected to the atoms N1-N4 were reoptimized.

Dihedral parameters

QM potential energy surface (PES) scans were calculated for dihedrals of porphyrin ring, dihedrals arising from the connection of axial and equatorial groups to the porphyrin ring (see Fig. S7). Dihedrals inside porphyrin ring were scanned by 50° in each direction with 10° increment, rotation of the ethoxy group around P-O bond were scanned by 180° in each direction with 15° increment, all other dihedrals were scanned by 90° in each direction with 15° increment. To reduce overfitting, we set to zero most of the dihedrals of porphyrin ring and dihedrals containing hydrogen atoms. We fit following dihedrals to the obtained PESs: rotation of the ethoxy group around P-O and O-C bond (CG321-OG301-PG0-NP3, PG0-OG301-CG321-CG331, etoxy dihedrals), dihedrals of the core ring (CPB-CPA-CPA-CPB, CPA-CPB-CP-CPB, rind dihedrals), and dihedrals arising from rotation and bending of the phenyl group relative to the core ring (CPB-CP-CG2R61-CG2R61, CP-CG2R61-CG2R61-CG2R61, phenyl dihedral). Ring dihedrals were fitted in **n(OH)$_2$** model and added to the **n(OEt)$_2$** model. The virtual annealing protocol designed by Guvench and MacKerell[2] and implemented in ffTK was used for optimization. Energy cut-off for fitting dihedrals force constant were set to 10 kcal/mol.

Figures S8-S10 illustrate comparison between QM and MM fitted dihedrals scans and Table 4 provides the information about root mean square error (RMSE). Although RMSE for Ring and Phenyl dihedrals are bigger than recommended value for CGenFF force field (~0.5 kcal/mol), the obtained RMSE are comparable with what was derive previously for similar structure[3] and low-energy configuration are reproduced within recommended RMSE.

Table S4. RMSE for fitted dihedrals.

Dihedrals	RMSE (kcal/mol)
Ring dihedrals	1.2
Ethyl dihedrals	0.36
Phenyl dihedrals	0.61

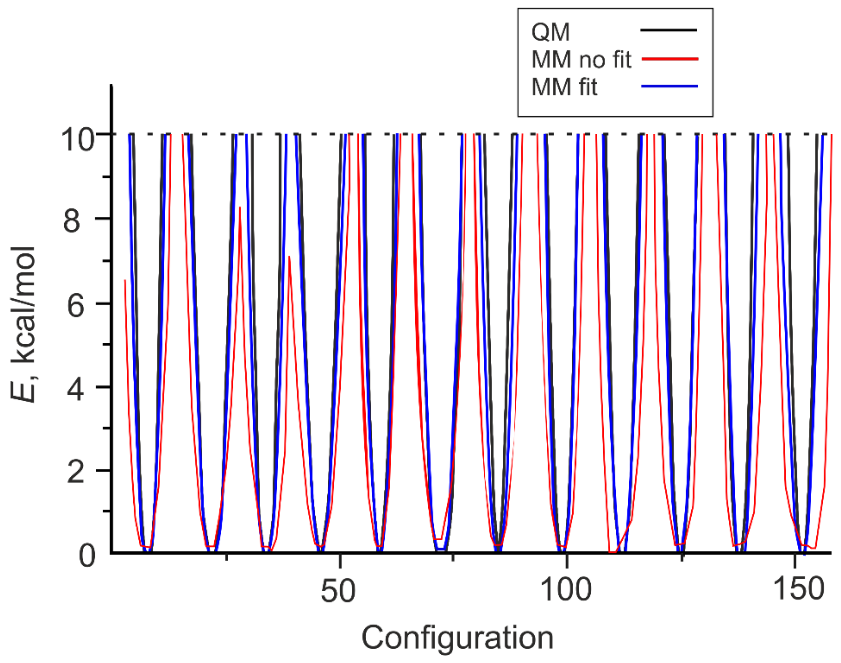

Figure S8. Comparison of QM and MM PESs for ring dihedrals.

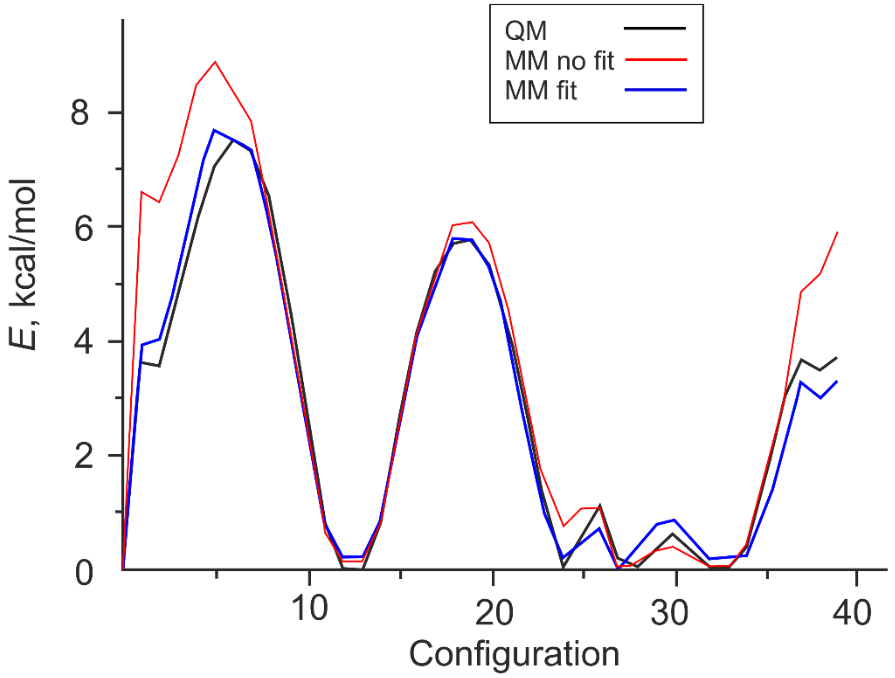

Figure S9. Comparison of QM and MM PESs for ethoxy dihedrals.

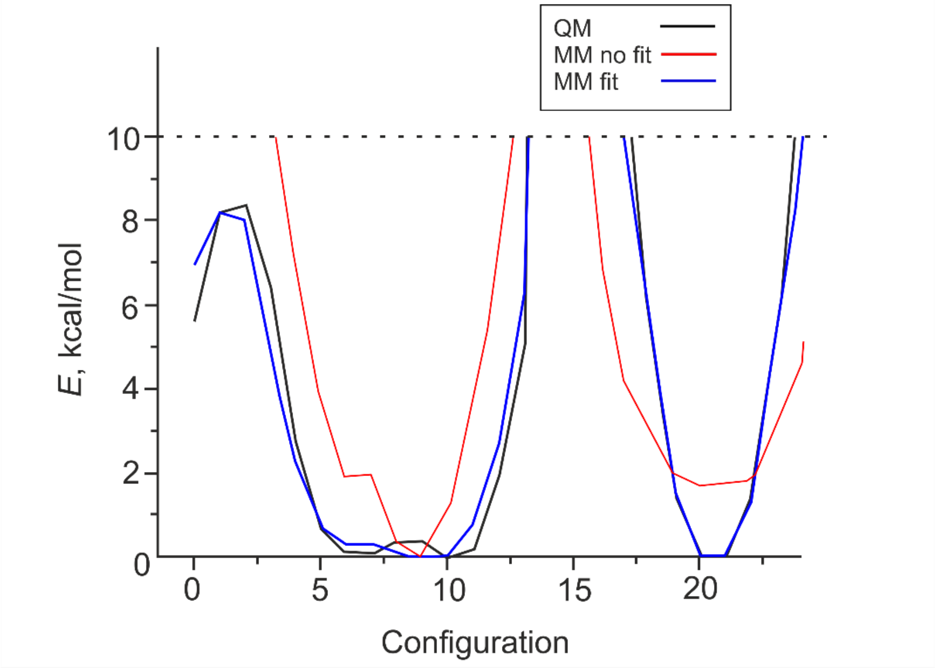

Figure S10. Comparison of QM and MM PESs for phenyl dihedrals.

Comparison of QM and MM optimized geometry

To elucidate the ability of our force field to reproduce the shape of porphyrin ring we compared QM and MM optimized geometry. The comparison between QM and MM optimized geometry is illustrated in Figure S11. MM-optimized geometry agrees well with geometry obtained from QM calculation. RMSD for both structures below 0.02 Å.

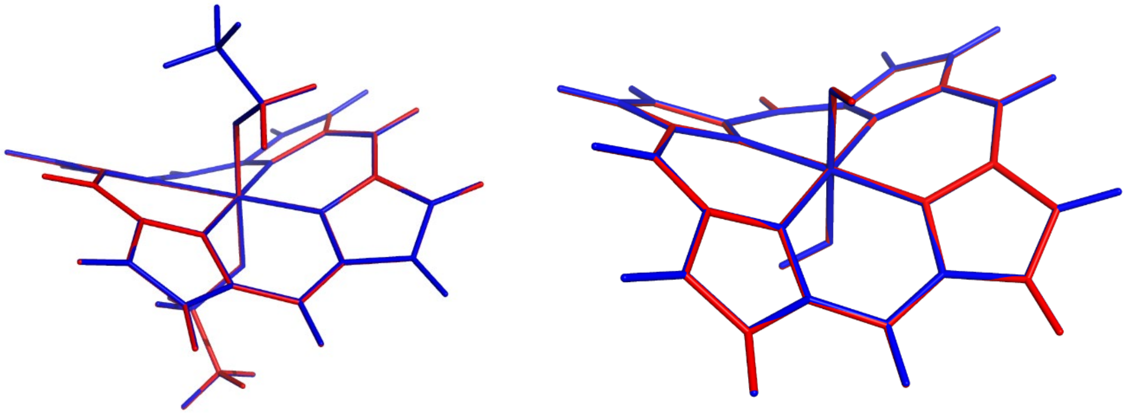

Figure S11. Comparison of QM (red) and MM (blue) optimized geometry.

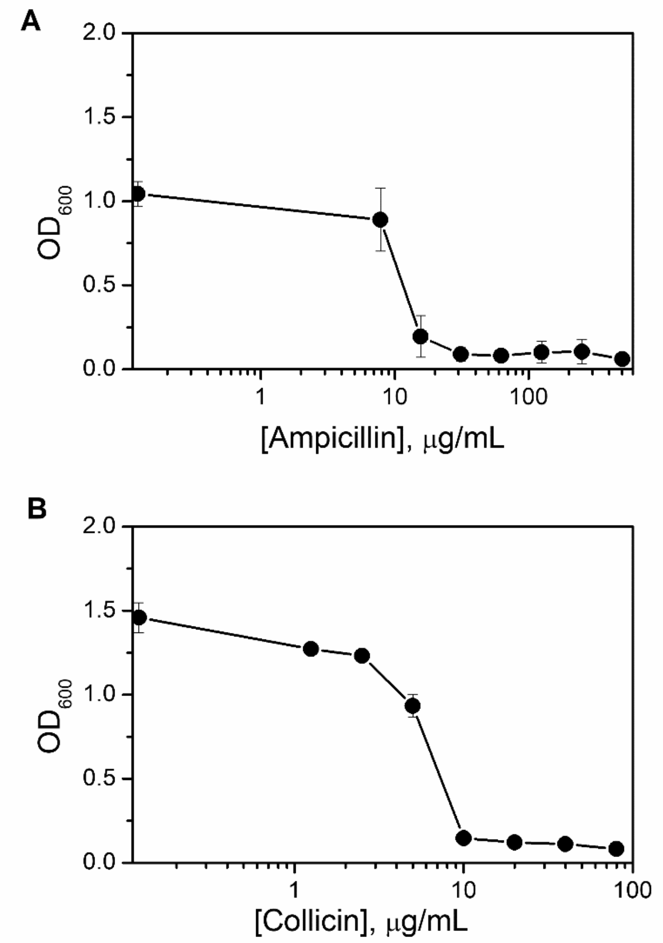

Figure S12. Dependence of the optical density at 600 nm (OD_{600}) for the bacterial suspension of *E. coli* incubated for 24 h with Ampicillin (**A**) and of *A. Baumannii* incubated for 24 h with Collicin (**B**). The study was carried out in 3 independent repetitions, the error was determined by ANOVA.

REFERENCES

(1) Mayne, C. G.; Saam, J.; Schulten, K.; Tajkhorshid, E.; Gumbart, J. C. Rapid Parameterization of Small Molecules Using the Force Field Toolkit. *J. Comput. Chem.* **2013**, *34* (32), 2757–2770. https://doi.org/10.1002/jcc.23422.

(2) Guvench, O.; MacKerell, A. D. Automated Conformational Energy Fitting for Force-Field Development. *J Mol Model* **2008**, *14* (8), 667–679. https://doi.org/10.1007/s00894-008-0305-0.

(3) Pavlova, A.; Parks, J. M.; Gumbart, J. C. Development of CHARMM-Compatible Force-Field Parameters for Cobalamin and Related Cofactors from Quantum Mechanical Calculations. *J. Chem. Theory Comput.* **2018**, *14* (2), 784–798. https://doi.org/10.1021/acs.jctc.7b01236.
